# Minimum Area Confidence Set Optimality for Simultaneous Confidence Bands for Percentiles With Applications to Drug Shelf‐Life Estimation

**DOI:** 10.1002/sim.70184

**Published:** 2025-09-09

**Authors:** Lingjiao Wang, Yang Han, Wei Liu, Frank Bretz

**Affiliations:** ^1^ Department of Mathematics University of Manchester Manchester UK; ^2^ School of Mathematical Sciences and Southampton Statistical Sciences Research Institute University of Southampton Southampton UK; ^3^ Novartis Pharma AG Basel Switzerland; ^4^ Section for Medical Statistics, Center for Medical Statistics, Informatics, and Intelligent Systems Medical University of Vienna Vienna Austria

**Keywords:** confidence set, drug stability study, multiple testing, percentile line, shelf‐life, simultaneous confidence band

## Abstract

One important property of any drug product is its stability over time. A key objective in drug stability studies is to estimate the shelf‐life of a drug, involving a suitable definition of the true shelf‐life and the construction of an appropriate estimate of the true shelf‐life. Simultaneous confidence bands (SCBs) for percentiles in linear regression are valuable tools for determining the shelf‐life in drug stability studies. In this paper, we propose a novel criterion, the Minimum Area Confidence Set (MACS) criterion, for finding the optimal SCB for percentile regression lines. This criterion focuses on the area of the constrained regions for the newly proposed pivotal quantities, which are generated from the confidence set for the unknown parameters of an SCB. We employ the new pivotal quantities to construct exact SCBs over any finite covariate intervals and use the MACS criterion to compare several SCBs of different forms. The optimal SCB under the MACS criterion can be used to construct the interval estimate of the true shelf‐life. Furthermore, a new computationally efficient method is proposed for calculating the critical constants of exact SCBs for percentile regression lines. A real data example on drug stability is provided for illustration.

## 1 Introduction

In drug development, a key property of any drug product is its stability over time. Drug stability studies are routinely conducted in order to measure the degradation of new drug products or existing drugs that have been reformulated or repackaged, as outlined by International Council of Harmonasation (ICH) guideline on stability testing of new drug substances and products; see ICH [1]. These studies provide patients and consumers with a high degree of confidence that a drug retains its strength, quality, and purity under appropriate storage conditions. Individual dosage units (e.g., tablets, capsules, vials) are sampled at predetermined time points and assayed for their active pharmaceutical ingredient (drug) content. The decline in drug content over time can be modeled based on these observations.

One important objective in drug stability studies is to estimate the true shelf‐life of a drug. The estimated shelf‐life is the storage period during which the potency of a drug product is expected to remain within the approved specification limit with an acceptable probability. The final day of the shelf life is known as the expiry date, which is required to be printed on the package label of the corresponding drug. According to ICH [2], the approved specification limits are no greater than ±10% of the labeled content of the drug. Current ICH guidelines, adopted by the U.S. Food and Drug Administration (FDA) and other major national regulatory agencies, recommend that the shelf‐life estimate should be based on an interval estimate of the mean change in drug content over storage time. Specifically, as outlined in ICH [1], the interval estimate of shelf‐life for a single batch is defined as the time interval during which the 1−α=95% confidence interval for the mean regression line of the observed drug content (y) and the time (x) crosses a pre‐specified level h of drug content, say h=98 (in percentage).

A commonly used statistical model for this purpose is simple linear regression of drug content (y) on the covariate time (x); see Kiermeier et al. [3], Quinlan et al. [4], and Liu et al. [5] for a review. Throughout this paper, we focus on linear regression for a single batch of drug, assuming that the drug content decreases over time, which results in a negative slope for the regression line. The situation where the drug content increases over time can be treated using a similar approach. To be specific, let the statistical model be $y=x^{T}\beta +\epsilon =\beta_{0}+\beta_{1}x+\epsilon$, where y denotes the observed drug content at time point x of the dosage unit, $x={\left(1,\;x\right)}^{T}$, $\beta ={\left(\beta_{0},\;\beta_{1}\right)}^{T}$, the random error ϵ follows a normal distribution $N(0,\;\sigma^{2})$, and $\beta_{0}$, $\beta_{1}$ and $\sigma^{2}$ are unknown parameters.

One problem in estimating the shelf‐life is deciding whether to construct a confidence interval for the average content of all dosage units, $x^{T}\beta$, or for the content of a γ proportion of all dosage units, $x^{T}\beta +z_{\gamma }\sigma$, with $z_{\gamma }$ denoting the 100γth percentile of standard normal distribution; see Ruberg and Stegeman [6]. According to the interval estimates of batch shelf‐life defined by ICH [1], the estimation is based on a (1−α)‐level confidence interval for the mean regression line, which implies that only half (i.e., 50%) of all the individual dosage units of the drug should have a drug content of at least h by the shelf‐life. However, from the patients' and consumers' point of view, it is expected that a larger proportion (e.g., 90%) of all the individual dosage units will have a drug content above the acceptable limit h by the expiry date. Quinlan et al. [4] also note that the ICH method can overestimate the true shelf‐life, making it inappropriate for ensuring drug safety.

Kiermeier et al. [3] consider an approximate (1−α)‐level pointwise confidence band (PCB) for the 100γth percentile regression line to provide an accurate estimate of the true shelf‐life. This PCB offers the inference of drug content for 100(1−γ)% of dose units at one specific time point. Liu et al. [5] provide a new method to address this problem by determining the shelf‐life using an exact (1−α)‐level PCB for the percentile line in linear regression.

Note that, the true shelf‐life can occur at any time point during the drug stability study period. Therefore, using simultaneous confidence bands (SCBs) to estimate the shelf‐life is a suitable approach, as the SCBs guarantee the confidence level 1−α of estimation at any time point within the covariate range of interest. SCBs for percentiles in linear regression are studied by Steinhorst and Bowden [7], Turner and Bowden [8, 9], and Thomas and Thomas [10] over the whole covariate range (−∞,∞). As the expiry date required by the regulatory to be printed on the package label of a drug often has a limit, it is reasonable to consider a finite interval of shelf‐life, that is, x∈(a,b). The specific duration of the expiration date varies depending on the drug product, its formulation, and stability testing results, but often a=0 and b=2 (in years) to justify an expiration dating period of over two years. Han et al. [11] construct exact SCBs over a finite covariate interval (a,b) and develop asymmetric SCBs for the 100γth percentile line, which could be used for more informative interval estimates of shelf‐life than the SCBs over (−∞,∞).

Han et al. [11] is the only available method for constructing exact symmetric and asymmetric SCBs. However, the pivotal quantities used in their approach for different types of SCBs vary in dimension, which complicates the implementation of their methods. In addition, they propose two methods for calculating the critical constants of SCBs: a simulation‐based method and a numerical quadrature method, both of which are computationally intensive.

As reviewed in Table 1, there are various forms of SCBs available in the statistical literature, and so it is desirable to identify the most informative SCB among different forms under a suitable criterion. Han et al. [11] determine the optimal SCB under the average width (AW) criterion [12]. The AW of a SCB is the average width between its upper and lower bounds over the covariate interval of interest (a,b), and has been widely used as an optimality criterion for comparing different forms of confidence bands; see Liu et al. [13]. It is noteworthy that the AW criterion has one limitation that it cannot directly provide the information of the unknown parameters β and σ when both parameters are of primary interest. Liu and Hayter [14] introduce the minimum area confidence set (MACS) criterion based on the area of the confidence set for β only, focusing on the SCBs for the mean regression line $x^{T}\beta$, see also Liu et al. [15] and Liu and Ah‐kine [16]. However, when estimating the shelf‐life, the SCBs for percentile lines are required, rather than the mean regression line, in order to assess whether a large proportion of dose units have a drug content above the threshold. In addition, note that making simultaneous inference for a percentile regression line over x∈(a,b) is equivalent to constructing the joint confidence set for β and σ. Therefore, this paper considers the MACS criterion based on the area of the confidence set for the unknown parameters β and σ. To date, no work in the literature has addressed such a MACS criterion for constructing and comparing SCBs for a percentile regression line.

**TABLE 1:** Six simultaneous confidence bands.

Name	Type	$\xi_{1}$	$\xi_{2}$	Origin
SB	I	1	0	Steinhorst and Bowden [7]
TBU	I	$\sqrt{\frac{2}{\nu }}\frac{\Gamma (\frac{\nu +1}{2})}{\Gamma (\frac{\nu }{2})}$	0	Turner and Bowden [8]
TBE	I	$\sqrt{\frac{2}{\nu }}\frac{\Gamma (\frac{\nu }{2})}{\Gamma (\frac{\nu -1}{2})}$	0	Turner and Bowden [8]
V	II	1	$1-\frac{2}{\nu }{\left(\frac{\Gamma (\frac{\nu +1}{2})}{\Gamma (\frac{\nu }{2})}\right)}^{2}$	Han et al. [11]
UV	II	$\sqrt{\frac{2}{\nu }}\frac{\Gamma (\frac{\nu +1}{2})}{\Gamma (\frac{\nu }{2})}$	$\frac{\nu }{2}{\left(\frac{\Gamma (\frac{\nu }{2})}{\Gamma (\frac{\nu +1}{2})}\right)}^{2}-1$	Han et al. [11]
TT	II	$\frac{4\nu -1}{4\nu }$	$\frac{1}{2\nu }$	Thomas and Thomas [10]

In the following, we construct exact (1−α)‐level symmetric and asymmetric two‐sided SCBs for the 100γth percentile line over any given covariate interval (a,b) to provide accurate interval estimates of shelf‐life. Note that both the lower and upper bounds are important for the patients, consumers, and manufacturers. Specifically, the lower bound of the interval estimate of the shelf‐life, based on the two‐sided SCBs, indicates that at least a (1−γ) proportion of all dosage units maintain a drug content above the limit h by this time point. The corresponding upper bound of the interval estimate of the shelf‐life represents the time point beyond which less than (1−γ) proportion of all dosage units retain a drug content above h. Hence, the two‐sided SCBs of our method can be applied to produce both upper or lower bounds for shelf‐life.

This paper makes three main contributions. First, we construct new pivotal quantities for the exact two‐sided (1−α)‐SCBs for the 100γth percentile line, $x^{T}\beta +z_{\gamma }\sigma$, over any given covariate interval x∈(a,b). Our newly proposed pivotal quantities link SCBs to the confidence set for the unknown parameters (β,σ) in linear regression. Furthermore, these pivotal quantities provide a more general approach for developing SCBs, making them applicable to all SCB types. Second, we develop the new MACS criterion to determine optimal SCBs that minimize the area of the confidence set for the unknown parameters (β,σ) by minimizing the area of the constrained regions for our new pivotal quantities. Third, we provide an efficient and exact method to compute the critical constants of SCBs for percentile lines. This new method uses numerical quadrature based on the newly proposed pivotal quantities. In comparison with the methods of Han et al. [11], it not only reduces the dimensions of the numerical integration but also eliminates the need for simulations, resulting in a significant reduction in computational costs.

Accordingly, this paper is organized as follows. In Section 2, we provide the details of the newly proposed pivotal quantities for the exact SCBs for percentile lines and the construction of the MACS criterion, including the numerical computation of the critical constants in Section 2.4. In Section 3, we report the results of a simulation study to assess the performances of different forms of SCBs, both symmetric and asymmetric, under the MACS criterion. In Section 4, we illustrate the proposed method with an illustrative example to determine the shelf‐life of a drug by using SCBs for percentile lines. Finally, Section 5 contains conclusions and discussions. The computational costs of our newly proposed method and the previous simulation‐based method, along with additional simulation results, are provided in the Supporting Information to save space.

## 2 Methodology

In this section, we focus on the construction of the MACS criterion for the exact (1−α)‐level SCBs for the 100γth percentile line over any pre‐specified interval of interest x∈(a,b).

### 2.1 Preliminaries

Consider the simple linear regression model

(1) $$y=x^{T}\beta +\epsilon =\beta_{0}+\beta_{1}x+\epsilon$$

where $x={\left(1,\;x\right)}^{T}$, $\beta ={\left(\beta_{0},\;\beta_{1}\right)}^{T}$, and the random error ϵ is assumed to follow a normal distribution $N(0,\;\sigma^{2})$. For a given dataset $ℰ=\{(x_{i},\;y_{i}),\;i=1,\;\ldots ,\;n\}$, let X denote the n×2 design matrix, the ith row of which is given by $x_{i}=(1,\;x_{i})$, i=1,2,…,n. Denote the least squares estimator of β and the unbiased estimator of $\sigma^{2}$ by $\hat{\beta }={\left({\hat{\beta }}_{0},\;{\hat{\beta }}_{1}\right)}^{T}={\left(X^{T}X\right)}^{-1}X^{T}y$ and ${\hat{\sigma }}^{2}=\|y-X\hat{\beta }{\|}^{2}/\nu$ with ν=n−2 and $y={\left(y_{1},\;\ldots ,\;y_{n}\right)}^{T}$. Then, we have $\hat{\beta }∼N_{2}(\beta ,\;\sigma^{2}{\left(X^{T}X\right)}^{-1})$, $\hat{\sigma }/\sigma ∼\sqrt{\chi_{\nu }^{2}/\nu }$ with the degrees of freedom ν, and $\hat{\beta }$ and $\hat{\sigma }$ are independent random variables. To facilitate the computation of critical constants in Section 2.2, we consider the mean‐centered design matrix without loss of generality.

In this paper, the true shelf‐life is defined as the time point $X_{\gamma }$, based on the 100γth percentile line $x^{T}\beta +z_{\gamma }\sigma$, that satisfies

$$\beta_{0}+\beta_{1}X_{\gamma }+z_{\gamma }\sigma =h$$

where $z_{\gamma }$ is the 100γth percentile of the standard normal distribution, and h is the pre‐specified acceptable limit of the drug content level. Hence, by the time point $X_{\gamma }$, no more than 100γ% (with γ=0.05) of all the individual dosage units will have a drug content below the acceptable limit h. It is noteworthy that the mean regression line $x^{T}\beta$ is the 50th percentile line, which is a special case of $x^{T}\beta +z_{\gamma }\sigma$ with γ=0.5.

The definition of the SCBs [l(x),u(x)], for the 100γth percentile line $x^{T}\beta +z_{\gamma }\sigma$, is given as follows.

Definition 1 A confidence band [l(x),u(x)], which satisfies

(2) $$\begin{array}{ll} & \;\underset{-\infty <\beta_{0},\;\beta_{1}<\infty ,\;\sigma >0}{\inf}P\left\{l(x)\leq x^{T}\beta +z_{\gamma }\sigma \leq u(x)\;\text{for all}\;x\in (a,\;b)\right\}=1-\alpha \; \end{array}$$

is called a (1−α)‐level simultaneous confidence band for the 100γth percentile line $x^{T}\beta +z_{\gamma }\sigma$, over a finite interval of interest x∈(a,b).

Consider two‐sided SCBs of the form

(3) $$\begin{array}{ll} & x^{T}\hat{\beta }+z_{\gamma }\hat{\sigma }⁄\xi_{1}-c_{1}\hat{\sigma }\sqrt{x^{T}{\left(X^{T}X\right)}^{-1}x+{\left(z_{\gamma }\right)}^{2}\xi_{2}}\leq x^{T}\beta +z_{\gamma }\sigma \\ & \;\leq x^{T}\hat{\beta }+z_{\gamma }\hat{\sigma }⁄\xi_{1}+c_{2}\hat{\sigma }\sqrt{x^{T}{\left(X^{T}X\right)}^{-1}x+{\left(z_{\gamma }\right)}^{2}\xi_{2}}\;\;\text{for all}\;x\in (a,\;b) \end{array}$$

where $c_{1}$ and $c_{2}$ are the critical constants that satisfy (2), and the constants $\xi_{1}\neq 0$ and $\xi_{2}$ are given and selected for constructing different forms of SCBs. The SCBs of the form (3) can be divided into symmetric and asymmetric based on whether $c_{1}=c_{2}$ or $c_{1}\neq c_{2}$, respectively, and into Type I and Type II based on whether $\xi_{2}=0$ or $\xi_{2}\neq 0$, respectively. For upper one‐sided SCBs, we have $c_{1}=\infty$, and for lower one‐sided SCBs, we have $c_{2}=\infty$.

Six different forms of SCBs have been published in the literature, with various $\left(\xi_{1},\;\xi_{2}\right)$‐values. Table 1 gives the values of $\xi_{1}$ and $\xi_{2}$ for these six bands: SB, TBU, TBE (Type I with $\xi_{2}=0$), V, UV and TT (Type II with $\xi_{2}\neq 0$). The corresponding asymmetric bands are denoted as SBa, TBUa, TBEa, Va, UVa, and TTa.

Han et al. [11] provide a two‐dimensional pivotal quantity for constructing the Type I band and a three‐dimensional pivotal quantity for constructing the Type II band. In this section, we construct a more general pivotal quantity that can be applied to both Type I and Type II bands in Table 1. All these six forms in Table 1 are compared under the MACS criterion in Section 3.

### 2.2 Pivotal Quantities and Confidence Sets

In this section, we construct the new pivotal quantities to provide a general way for developing the SCBs in Table 1, regardless of their types. The constrained regions for the new pivotal quantities and the confidence sets for unknown parameters $\left(\beta_{0},\;\beta_{1},\;\sigma \right)$ are also provided based on the corresponding SCBs.

Without loss of generality, we use the mean‐centered covariate values to fit the regression in (1). Then, the covariate x in (1) is replaced by $\left(x-\bar{x}\right)$, where $\bar{x}=\frac{1}{n}\sum_{i=1}^{n}x_{i}$ denotes the mean of $x_{i}$‐values in the training dataset $ℰ=\{(x_{i},\;y_{i}),\;i=1,\;\ldots ,\;n\}$. Accordingly, the vector x in (1) becomes $x={\left(1,\;x-\bar{x}\right)}^{T}$, and the ith row of design matrix X becomes $\left(1,\;x_{i}-\bar{x}\right)$. Let $S_{xx}=\sum_{i=1}^{n}{\left(x_{i}-\bar{x}\right)}^{2}$, then ${\left(X^{T}X\right)}^{-1}=\left(\begin{array}{ll} 1/n & 0\\ 0 & 1/S_{xx} \end{array}\right)$ is a 2×2 diagonal matrix. Let P be the square root matrix of ${\left(X^{T}X\right)}^{-1}$ given by

$$P={\left(X^{T}X\right)}^{-1/2}=\left(\begin{array}{cc} \frac{1}{\sqrt{n}} & 0\\ 0 & \frac{1}{\sqrt{S_{xx}}} \end{array}\right)$$

and $Px={\left(\frac{1}{\sqrt{n}},\;\frac{x-\bar{x}}{\sqrt{S_{xx}}}\right)}^{T}$. From (3), the confidence level of a two‐sided SCB is given by

(4) $$\begin{array}{ll} & P\left\{x^{T}\hat{\beta }+z_{\gamma }\hat{\sigma }⁄\xi_{1}-c_{1}\hat{\sigma }\sqrt{x^{T}{\left(X^{T}X\right)}^{-1}x+{\left(z_{\gamma }\right)}^{2}\xi_{2}}\leq x^{T}\beta +z_{\gamma }\sigma \right.\\ & \;\left.\leq x^{T}\hat{\beta }+z_{\gamma }\hat{\sigma }⁄\xi_{1}+c_{2}\hat{\sigma }\sqrt{x^{T}{\left(X^{T}X\right)}^{-1}x+{\left(z_{\gamma }\right)}^{2}\xi_{2}}\;\text{for all}\;x\in (a,\;b)\right\}\\ & \;=P\left\{-c_{2}\leq \frac{{\left(Px\right)}^{T}N⁄U+z_{\gamma }(1⁄\xi_{1}-1⁄U)}{\sqrt{{\left(Px\right)}^{T}(Px)+{\left(z_{\gamma }\right)}^{2}\xi_{2}}}\leq c_{1}\;\text{for all}\;x\in (a,\;b)\right\}\\ & \;=P\left\{-c_{2}\leq \frac{\left(\frac{N_{1}}{\sqrt{n}}+\frac{N_{2}(x-\bar{x})}{\sqrt{S_{xx}}}\right)⁄U+z_{\gamma }(1⁄\xi_{1}-1⁄U)}{\sqrt{\frac{1}{n}+\frac{{\left(x-\bar{x}\right)}^{2}}{S_{xx}}+z_{\gamma }^{2}\xi_{2}}}\leq c_{1}\;\text{for}\text{all}\;x\in (a,\;b)\right\} \end{array}$$

where $N:={\left(N_{1},\;N_{2}\right)}^{T}=P^{-1}(\hat{\beta }-\beta )/\sigma ∼N_{2}(0,\;I_{2})$, and $U:=\hat{\sigma }/\sigma ∼\sqrt{\chi_{\nu }^{2}/\nu }$.

Let $w={\left(\sqrt{\frac{1}{n}+z_{\gamma }^{2}\xi_{2}},\;\frac{x-\bar{x}}{\sqrt{S_{xx}}}\right)}^{T}$. Define the new pivotal quantity

(5) $$K=\left(\begin{array}{c} K_{1}\\ K_{2} \end{array}\right)=\left(\begin{array}{c} \frac{N_{1}-z_{\gamma }\sqrt{n}}{U\sqrt{1+nz_{\gamma }^{2}\xi_{2}}}+\frac{z_{\gamma }\sqrt{n}}{\xi_{1}\sqrt{1+nz_{\gamma }^{2}\xi_{2}}}\\ N_{2}/U \end{array}\right)$$

and a region of K, denoted as $R_{K}$, by

(6) $$R_{K}=\left\{K:-c_{2}\leq \frac{w^{T}K}{\left\|w\right\|}\leq c_{1}\;\text{for all}\;x\in (a,\;b)\right\}\subset {ℛ}^{2}.$$

Then, in general, the critical constants $\left(c_{1},\;c_{2}\right)$ for all (1−α)‐level SCBs in Table 1 satisfy that $P\{K\in R_{K}\}=1-\alpha$.

Next, we develop the confidence sets for unknown parameters $\theta ={\left(\beta_{0},\beta_{1},\sigma \right)}^{T}$ using the region $R_{K}$ in (6). Let $a={\left(\frac{z_{\gamma }\sqrt{n}}{\xi_{1}\sqrt{1+nz_{\gamma }^{2}\xi_{2}}},\;0\right)}^{T}$, $b={\left({\hat{\beta }}_{0},\;{\hat{\beta }}_{1},\;0\right)}^{T}$, $M=\left(\begin{array}{ll} \sqrt{\frac{1}{1+nz_{\gamma }^{2}\xi_{2}}} & 0\\ 0 & 1 \end{array}\right)$ and $U=\frac{1}{\hat{\sigma }}\left(\begin{array}{lll} \sqrt{n} & 0 & z_{\gamma }\sqrt{n}\\ 0 & \sqrt{S_{xx}} & 0 \end{array}\right)$. Then the confidence set for unknown parameters $\theta ={\left(\beta_{0},\beta_{1},\sigma \right)}^{T}$ is given by

(7) $$C_{\theta }=\left\{\theta :\;K\in R_{K}\right\}=\left\{\theta :\;MU(b-\theta )+a\in R_{K}\right\}\subset {ℛ}^{3};$$

the detailed derivation is provided in Appendix A. The new criterion is based on the area of the confidence set $C_{\theta }$ for θ. The smaller the area of $C_{\theta }$, the better and more informative the corresponding SCB. Intuitively, each θ in a confidence set corresponds to one 100γth percentile line $x^{T}\beta +z_{\gamma }\sigma$ that lies completely inside the corresponding SCB and vice versa. Note that the pivotal quantity K is generated from the parameter θ via the transformation K=MU(b−θ)+a. It is clear that the distribution of K depends on the values of $\left(\xi_{1},\;\xi_{2}\right)$ via the shift a and scaling M. Consequently, when comparing the SCBs in Table 1 with various $\left(\xi_{1},\;\xi_{2}\right)$‐values, one cannot conclude that a smaller area of $R_{K}$ necessarily implies a smaller confidence set $C_{\theta }$ for unknown parameter θ.

Next, we develop a new pivotal quantity, defined as $T=U(b-\theta )={\left(\frac{N_{1}-z_{\gamma }\sqrt{n}}{U},\;\frac{N_{2}}{U}\right)}^{T}$, which does not depend on the values of $\left(\xi_{1},\;\xi_{2}\right)$. Also, we define the region $R_{T}$ by

$$R_{T}=\left\{T:\;MT+a\in R_{K}\right\}=\left\{T:\;T\in M^{-1}R_{K}-M^{-1}a\right\}\subset {ℛ}^{2}.$$

Then, the confidence set $C_{\theta }$ in (7) can further be represented as

$$C_{\theta }=\left\{\theta :\;MT+a\in R_{K}\right\}=\{\theta :\;T\in R_{T}\}\subset {ℛ}^{3}.$$

Since the transformation T=U(b−θ) is independent of $\left(\xi_{1},\;\xi_{2}\right)$‐values, then a smaller area of $R_{T}$ corresponds to a smaller confidence set $C_{\theta }$ for unknown parameter θ. Hence, we measure the volume of the confidence set $C_{\theta }$ in terms of the area of $R_{T}$, which is given in the theorem below.

Theorem 1 *The area of* $R_{T}$ *is given in terms of the area of* $R_{K}$ *by*

(8) $$\text{Area}(R_{T})=|M^{-1}|\text{Area}(R_{K})=\text{Area}(R_{K})\sqrt{1+nz_{\gamma }^{2}\xi_{2}}$$

*where* Area(C) *denotes the area of set* C.

By using matrix scaling and geometric scaling, we have

$$\begin{array}{ll} \text{Area}(R_{T}) & =|M^{-1}|\text{Area}(R_{K})=\text{Area}(R_{K}){\left|\left(\begin{array}{cc} \sqrt{\frac{1}{1+nz_{\gamma }^{2}\xi_{2}}} & 0\\ 0 & 1 \end{array}\right)\right|}^{-1}\\ & =\text{Area}(R_{K})\sqrt{1+nz_{\gamma }^{2}\xi_{2}}\;. \end{array}$$

### 2.3 MACS Criterion

In this section, we propose and discuss the new optimality criterion, the MACS criterion, based on our newly introduced pivotal quantities K and T.

**Criterion**: For given values of γ, n, the design matrix X and the covariate interval of interest (a,b) in (3), the optimal (1−α)‐level SCB has the smallest $\text{Area}(R_{T})$ in (8) among all possible regions satisfying $P\{K\in R_{K}\}=1-\alpha$.

In order to determine the optimal SCB among all possible forms given in Table 1, the area of $R_{T}$, $\text{Area}(R_{T})$, for each SCB should be computed and compared. Since $\text{Area}(R_{T})$ depends on $\text{Area}(R_{K})$, as shown in Theorem 1, we next focus on deriving $\text{Area}(R_{K})$ using the polar coordinates of the pivotal quantity K.

**FIGURE 1:**
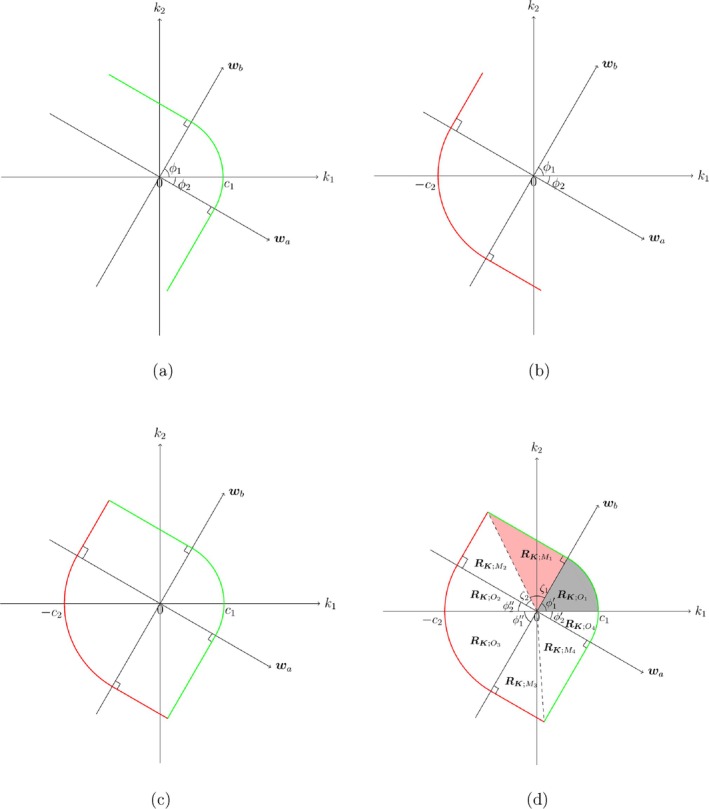
(a–c) The construction of $R_{K}$; (d) The partitioned regions of $R_{K}$. (a) The right boundary of $R_{K}$, denoted by $\left\{K:\frac{w^{T}K}{\left\|w\right\|}\leq c_{1}\;\text{for all}\;x\in (a,\;b)\right\}$. (b) The left boundary of $R_{K}$, denoted by $\left\{K:-c_{2}\leq \frac{w^{T}K}{\left\|w\right\|}\;\text{for all}\;x\in (a,\;b)\right\}$. (c) The area of $R_{K}$, denoted by $\left\{K:-c_{2}\leq \frac{w^{T}K}{\left\|w\right\|}\leq c_{1}\;\text{for all}\;x\in (a,\;b)\right\}$. (d) The partitioned regions of $R_{K}$ by using polar coordinates of K.

The constraints in (6) restrict K to the lemon‐shaped region $R_{K}$ in Figure 1c, bounded by two thick curved lines $\left\{K:\frac{w^{T}K}{\left\|w\right\|}\leq c_{1}\;\text{for all}\;x\in (a,\;b)\right\}$ given in Figure 1a and $\left\{K:-c_{2}\leq \frac{w^{T}K}{\left\|w\right\|}\;\text{for all}\;x\in (a,\;b)\right\}$ given in Figure 1b. The angle $\phi =\phi_{1}+\phi_{2}$ is formed by the boundary vectors of w over x∈(a,b), where the boundary vectors are given by $w_{a}={\left(\sqrt{\frac{1}{n}+z_{\gamma }^{2}\xi_{2}},\;\frac{a-\bar{x}}{\sqrt{S_{xx}}}\right)}^{T}$ and $w_{b}={\left(\sqrt{\frac{1}{n}+z_{\gamma }^{2}\xi_{2}},\;\frac{b-\bar{x}}{\sqrt{S_{xx}}}\right)}^{T}$. Then, ϕ is calculated from

(9) $$\begin{array}{ll} \cos(\phi ) & =\frac{\frac{1}{n}+z_{\gamma }^{2}\xi_{2}+\frac{\left(a-\bar{x})(b-\bar{x}\right)}{S_{xx}}}{\sqrt{\left[\frac{1}{n}+z_{\gamma }^{2}\xi_{2}+\frac{{\left(a-\bar{x}\right)}^{2}}{S_{xx}}\right]\left[\frac{1}{n}+z_{\gamma }^{2}\xi_{2}+\frac{{\left(b-\bar{x}\right)}^{2}}{S_{xx}}\right]}}\;. \end{array}$$

In Figure 1, ϕ∈(0,π) increases when (b−a) increases, and the critical constants $c_{1}$ and $c_{2}$ can be determined from the equation $P\{K\in R_{K}\}=1-\alpha$. Note that the set $R_{K}$ in Figure 1c is partitioned into four triangles $R_{K;M_{i}}$ and four fans $R_{K;O_{i}}$ (i=1,2,3,4); see Figure 1d. Thus, the set $R_{K}$ can be expressed as

$$R_{K}{=⋃}_{i=1}^{4}\left(R_{K;M_{i}}\cup R_{K;O_{i}}\right).$$

Based on this expression of $R_{K}$, we can simplify the calculation of the confidence level $P\{K\in R_{K}\}$ for SCBs in Table 1 by using the polar coordinates of K as shown next.

Let (R,δ) be the polar coordinates of $K={\left(K_{1},\;K_{2}\right)}^{T}$, i.e., $K_{1}=R\cos\delta$ and $K_{2}=R\sin\delta$, where R=‖K‖>0 and δ∈[0,2π) is the angle between the vectors ${\left(1,\;0\right)}^{T}$ and K. The joint density of (R,δ) is given by

(10) $$\begin{array}{ll} f_{R,\delta }(r,\delta ) & =f_{K_{1},K_{2}}(k_{1},k_{2})r\\ & =\frac{r\exp(-q_{3}^{2}/2)\nu^{\nu /2}}{q_{1}2^{\nu /2}\pi \Gamma (\nu /2)}\int_{0}^{\infty }k_{3}^{\nu +1}\\ & \;\times \exp\left\{-\frac{1}{2}\left[\left({\left(\frac{r\cos\delta -q_{2}}{q_{1}}\right)}^{2}+r^{2}{\left(\sin\delta \right)}^{2}+\nu \right)k_{3}^{2}\right.\right.\\ & \;\left.\left.+\frac{2q_{3}(r\cos\delta -q_{2})}{q_{1}}k_{3}\right]\right\}dk_{3}\; \end{array}$$

where ν=n−2, $q_{1}=\sqrt{\frac{1}{1+nz_{\gamma }^{2}\xi_{2}}}$, $q_{2}=\frac{z_{\gamma }\sqrt{n}}{\xi_{1}\sqrt{1+nz_{\gamma }^{2}\xi_{2}}}$ and $q_{3}=z_{\gamma }\sqrt{n}$; the derivation of $f_{K_{1},K_{2}}(k_{1},k_{2})$ in (10) is given in Appendix C.

The theorem below gives an expression of the confidence level of an SCB using (10) and Figure 1d.

Theorem 2 *For given* $c_{1}$ *and* $c_{2}$, *the confidence level of the SCB can be expressed as*:

$$\begin{array}{ll} & P\left\{x^{T}\hat{\beta }+z_{\gamma }\hat{\sigma }⁄\xi_{1}-c_{1}\hat{\sigma }\sqrt{x^{T}{\left(X^{T}X\right)}^{-1}x+{\left(z_{\gamma }\right)}^{2}\xi_{2}}\leq x^{T}\beta +z_{\gamma }\sigma \right.\\ & \left.\;\leq x^{T}\hat{\beta }+z_{\gamma }\hat{\sigma }⁄\xi_{1}+c_{2}\hat{\sigma }\sqrt{x^{T}{\left(X^{T}X\right)}^{-1}x+{\left(z_{\gamma }\right)}^{2}\xi_{2}}\;\text{for all}\;x\in (a,\;b)\right\}\\ & \;=P\{K\in R_{K}\}\\ & \;=P\left\{(R,\;\delta )\in {⋃}_{i=1}^{4}\left(R_{K;M_{i}}\cup R_{K;O_{i}}\right)\right\}\\ & \;=\sum_{i=1}^{4}\left(P\left\{(R,\;\delta )\in R_{K;M_{i}}\right\}+P\left\{(R,\;\delta )\in R_{K;O_{i}}\right\}\right)\\ & \;=\sum_{i=1}^{4}\left(\int \int_{(R,\delta )\in R_{K;M_{i}}}f_{R,\delta }(r,\delta )drd\delta \right.\left.+\int \int_{(R,\delta )\in R_{K;O_{i}}}f_{R,\delta }(r,\delta )drd\delta \right). \end{array}$$

The proof of Theorem 2 is given in Appendix D. From Theorems 1 and 2, for a given type of SCBs in (3) with given ($\xi_{1}$, $\xi_{2}$), γ, design matrix X and covariate interval (a,b), the critical constants $\left(c_{1},\;c_{2}\right)$ of the optimal SCB under the MACS criterion can be determined by minimizing the $\text{Area}(R_{T})$ in (8) subject to the constraint $P\{K\in R_{K}\}=1-\alpha .$

### 2.4 Computation of the Critical Constants $c_{1}$ and $c_{2}$

In this section, we provide a general algorithm for computing the critical constants $\left(c_{1},\;c_{2}\right)$ using the polar coordinates (R,δ) of pivotal quantity K and Theorem 2. For a symmetric SCB with $c_{1}=c_{2}=c$, determining the critical constant c can be simplified to a root‐finding problem, that is, computing the unique root c that satisfies $P\{K\in R_{K}\}{|}_{c_{1}=c_{2}=c}=1-\alpha$ according to Theorem 2. Note that a symmetric SCB is just a special case of the SCB in (3). Hence, the critical constant c for the symmetric SCB can be used as the starting point for searching the critical constants $\left(c_{1},\;c_{2}\right)$ of the corresponding optimal MACS asymmetric SCB with $c_{1}\neq c_{2}$. It is noteworthy that if γ≤0.5 then $c_{1}\geq c$, and if γ≥0.5 then $c_{2}\geq c$. We use Algorithm 1 for computing the $\left(c_{1},\;c_{2}\right)$ of the optimal MACS asymmetric SCBs.

ALGORITHM 1 **Compute the $\left(c_{1},\;c_{2}\right)$ of the optimal MACS asymmetric SCB.**

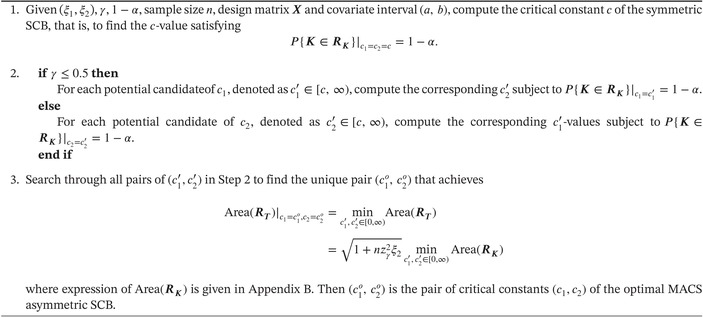

Han et al. [11] compare the SCBs in Table 1 under the AW criterion. For given values of γ, n, the design matrix X and the covariate interval of interest (a,b) in (3), the optimal (1−α)‐level SCB has the smallest AW, where

(11) $$\text{AW}=\frac{1}{b-a}\int_{a}^{b}(c_{1}+c_{2})\hat{\sigma }\sqrt{x^{T}{\left(X^{T}X\right)}^{-1}x+{\left(z_{\gamma }\right)}^{2}\xi_{2}}dx.$$

Han et al. [11] propose two methods for computing the critical constants $\left(c_{1},\;c_{2}\right)$: a numerical quadrature method and a simulation‐based method. Next, we illustrate how the computational methods in Han et al. [11] are more time‐consuming than the method of this paper in Algorithm 1.

For the numerical quadrature method of Han et al. [11], the critical constants are computed exactly by solving equations that involve three‐dimensional integrations and indicator functions, given by

$$\begin{array}{ll} & \;\int_{0}^{\infty }\int_{-\infty }^{\infty }\int_{-\infty }^{\infty }f(n_{1},n_{2},u)I_{\left\{-c_{2}\leq \frac{\left(\frac{N_{1}}{\sqrt{n}}+\frac{N_{2}(x-\bar{x})}{\sqrt{S_{xx}}}\right)⁄U+z_{\gamma }(1⁄\xi_{1}-1⁄U)}{\sqrt{\frac{1}{n}+\frac{{\left(x-\bar{x}\right)}^{2}}{S_{xx}}+z_{\gamma }^{2}\xi_{2}}}\;\leq \;c_{1}\;\text{for all}\;x\in (a,b)\right\}}dn_{1}dn_{2}du=1-\alpha \end{array}$$

where $f(n_{1},n_{2},u)$ is the joint density function of $\left(N_{1},\;N_{2},\;U\right)$, and $I_{A}$ denotes the indicator function of the set A. Due to the three‐dimensional numerical quadrature involved, it takes substantially longer computation time than the method of this paper, which uses only two‐dimensional numerical quadrature.

For the simulation‐based method of Han et al. [11], they first generate L independent replicates of (N,U): $\left(N_{i},\;U_{i}\right)$ for i=1,…,L, and then compute the corresponding $\left(c_{1;i},\;c_{2;i}\right)$‐values. Finally, the pair $\left(c_{1},\;c_{2}\right)$ for an asymmetric SCB is selected such that it not only satisfies the confidence level 1−α but also minimizes the AW in (11). The computational costs are reduced considerably by using this simulation‐based method in comparison with their numerical quadrature method. However, L=1000000 iterations are required to ensure the critical constants are accurate to at least two decimal places.

In contrast, the new method does not involve a Monte Carlo simulation and takes only a few seconds to compute the critical constants. This enables the dynamic construction of SCBs to be performed efficiently.

To highlight the improvements in computational efficiency, we conducted a simulation study to compare our new method with the simulation‐based approach. Since the numerical quadrature method is even more time‐consuming than the simulation‐based method, we do not compare our new method with the numerical quadrature method in this simulation study. Our numerical results show that the computation of critical constants using our newly proposed method takes about 20 s for symmetric SCBs and about 200 s for asymmetric SCBs on an ordinary Windows PC (Intel(R) Core(TM) i7‐6700 CPU with 3.40 GHz, 3.41 GHz, RAM 16.0 GB). In contrast, the simulation‐based method in Han et al. [11] takes over $8\times 10^{2}$ s for symmetric SCBs and no less than $4\times 10^{3}$ s for asymmetric SCBs on the same Windows PC, using 1 000 000 simulations. This decrease in time by about one magnitude demonstrates that our newly proposed method is significantly more efficient. Detailed computational costs for various scenarios are provided in the Supporting Information.

## 3 Simulation Study for Evaluating the Performance of SCBs

In our numerical comparisons, we assess the performance of symmetric and asymmetric SCBs within each type (Type I bands with $\xi_{2}=0$ and Type II bands with $\xi_{2}\neq 0$), as well as compare the performance across Type I and Type II bands, in various scenarios.

### 3.1 Design of Simulation Study

Note that the critical constants $\left(c_{1},\;c_{2}\right)$ for SCBs and the areas of regions $R_{K}$ in (6) depend on the design matrix X, the interval (a,b), n, γ, and 1−α. Based on this, we design the simulation study as follows. Given the data $ℰ=\{(x_{i},\;y_{i}),\;i=1,\;\ldots ,\;n\}$, we have the mean $\bar{x}=\frac{1}{n}\sum_{i=1}^{n}x_{i}$ and the sum of squares $S_{xx}=\sum_{i=1}^{n}{\left(x_{i}-\bar{x}\right)}^{2}$. Here, we focus on the case where $a-\bar{x}=-(b-\bar{x})$, i.e., the interval x∈(a,b) is symmetric around $\bar{x}$. Let $s=\frac{b-\bar{x}}{\sqrt{S_{xx}}}=-\frac{a-\bar{x}}{\sqrt{S_{xx}}}$. Recall $w={\left(\sqrt{\frac{1}{n}+z_{\gamma }^{2}\xi_{2}},\;\frac{x-\bar{x}}{\sqrt{S_{xx}}}\right)}^{T}$ in (6). The $\text{Area}(R_{K})$ then depends on the value of $\frac{x-\bar{x}}{\sqrt{S_{xx}}}$, which lies in the interval $\left(\frac{a-\bar{x}}{\sqrt{S_{xx}}},\;\frac{b-\bar{x}}{\sqrt{S_{xx}}}\right)=(-s,\;s)$ and is symmetric about 0. Note that $s=\frac{b-a}{2\sqrt{S_{xx}}}$, then the value of (b−a) increases as s increases. Thus, the critical constants $\left(c_{1},\;c_{2}\right)$ depend on the design matrix X and the covariate interval (a,b) via s. In this case, the critical constants $\left(c_{1},\;c_{2}\right)$ and the areas of regions $R_{K}$ depend only on s, n, γ, and 1−α.

In total, 12 different exact SCBs are considered under 24 different scenarios. The 12 SCBs include both the symmetric and asymmetric bands of the six forms given in Table 1. The 24 scenarios are derived from different combinations of the confidence level 1−α, percentile γ, sample size n, and value of s. In our numerical comparison, the settings we used are as follows: (i) confidence level: 1−α=0.90,0.99; (ii) percentile: γ=0.75,0.95; (iii) sample size: n=10,100; and (iv) s=0.1,1,10.

### 3.2 Measuring Performance of SCBs

The main goal of the simulation study is to identify the optimal form of SCBs under the MACS criterion. To make the evaluation more concrete and quantifiable, we assess the performance of different SCBs by comparing the ratios of areas of the regions $R_{T}$.

Suppose two bands A and B have regions $R_{T;A}$ and $R_{T;B}$, respectively, for given values of (a,b), n, α, γ, $\xi_{1}$ and $\xi_{2}$. Clearly the ratio

(12) $$r=\frac{\text{Area}(R_{T;B})}{\text{Area}(R_{T;A})}$$

is of interest for comparing bands A and B under the MACS criterion. When r>1, the area of $R_{T;A}$ is smaller and the corresponding confidence set for unknown parameter θ is smaller, and so A is better than B; otherwise, B is better than A.

### 3.3 Simulation Results

The results of the simulation study are summarized here using the r‐ratio in (12). According to (9), the ϕ‐values for each SCB depend on (a,b) and X only through s. For Type I bands, the ϕ‐values are the same because $\xi_{2}$ is fixed at $\xi_{2}=0$. However, for Type II bands, the ϕ‐values depend on different $\xi_{2}$‐values. Even with different $\xi_{2}$‐values, the ϕ‐values for all Type II bands remain the same up to two decimal places for given s and n. In Table 2, $\phi^{I}$ and $\phi^{II}$ denote the ϕ‐values for Type I and Type II bands, respectively.

**TABLE 2:** Ratios r, relative to UVa, of $\text{Area}(R_{T})$ for SCBs: TBE, TBEa, UV.

1−α	γ	n	s	$\phi^{I}$	$\phi^{II}$	TBE	TBEa	UV	UVa
0.90	0.75	10	0.1	0.613	0.543	1.003	1.003	1.020	1
			1	2.529	2.451	1.007	1.004	1.023	1
			10	3.078	3.070	1.003	0.998	1.020	1
		100	0.1	1.571	1.466	1.012	1.010	1.001	1
			1	2.942	2.920	1.006	1.006	1.001	1
			10	3.122	3.119	1.006	1.006	1.001	1
	0.95	10	0.1	0.613	0.543	1.014	1.013	1.081	1
			1	2.529	2.451	1.124	1.118	1.090	1
			10	3.078	3.070	1.098	1.084	1.071	1
		100	0.1	1.571	1.466	1.087	1.087	1.003	1
			1	2.942	2.920	1.112	1.111	1.003	1
			10	3.122	3.119	1.112	1.107	1.003	1
0.99	0.75	10	0.1	0.613	0.543	1.211	1.004	1.286	1
			1	2.529	2.451	1.155	0.992	1.172	1
			10	3.078	3.070	1.126	0.968	1.143	1
		100	0.1	1.571	1.466	1.033	1.019	1.021	1
			1	2.942	2.920	1.023	1.007	1.015	1
			10	3.122	3.119	1.022	1.005	1.015	1
	0.95	10	0.1	0.613	0.543	1.581	1.017	1.731	1
			1	2.529	2.451	1.770	1.056	1.464	1
			10	3.078	3.070	1.618	0.906	1.317	1
		100	0.1	1.571	1.466	1.197	1.144	1.070	1
			1	2.942	2.920	1.267	1.179	1.046	1
			10	3.122	3.119	1.264	1.162	1.044	1

*Note:* $\phi^{I}$ is the angle ϕ in (9) for Type I bands with $\xi_{2}=0$ (TBE and TBEa). $\phi^{II}$ is the angle ϕ in (9) for Type II bands with $\xi_{2}\neq 0$ (UV and UVa).

Based on our numerical results within each type, asymmetric SCBs have smaller areas of $R_{T}$ and are better than symmetric SCBs, for both Type I and Type II bands. For Type I bands, asymmetric SCBs (SBa, TBUa, TBEa) outperform symmetric SCBs (SB, TBU, TBE) by up to about 100% when both 1−α and γ are large. Among the three symmetric Type I bands, TBE has the smallest area of $R_{T}$, and so TBE is the best in terms of MACS. The difference in the areas of $R_{T}$ among the three asymmetric Type I bands is small. For Type II bands, asymmetric SCBs (Va, TTa, UVa) are better than symmetric SCBs (V, TT, UV), by as much as about 80% when 1−α is large and s is small. Among the three symmetric Type II bands, the V band consistently performs slightly worse than the UV band under the MACS criterion, and therefore, the V band is not recommended. The differences in the areas of $R_{T}$ between UV and TT are small, so either band can be used. Additionally, the differences in the areas of $R_{T}$ among the three asymmetric Type II bands are small. In general, an asymmetric SCB is better than the corresponding symmetric SCB according to the MACS criterion. The detailed numerical results are provided in the Supporting Information.

In the comparative analysis across Type I and Type II bands, we focus on the performance of the TBE, TBEa, UV, and UVa bands under the MACS criterion, as TBE and UV are the best Type I band and Type II band, respectively. Table 2 presents the r‐ratios of $\text{Area}(R_{T})$ for TBE, TBEa, and UV relative to UVa. Note that the r‐ratios for symmetric bands are always larger than one, indicating that the asymmetric bands (TBEa and UVa) outperform the symmetric bands (TBE and UV). When n=100, UVa is the best, with the $\text{Area}(R_{T})$ for UVa consistently smaller than that for TBEa. When n=10, UVa is the best in most cases, while TBEa performs best if the x‐values in the training dataset are dispersed (i.e., n=10 and s=10).

**FIGURE 2:**
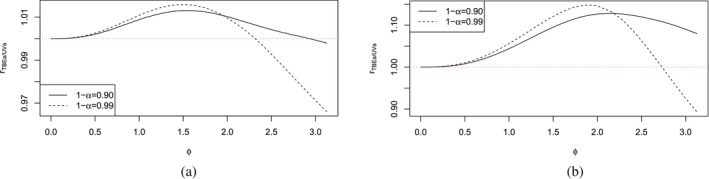
The ratios $r=\frac{\text{Area}(R_{T;\text{TBEa}})}{\text{Area}(R_{T;\text{UVa}})}$ of $\text{Area}(R_{T})$ for TBEa relative to UVa, with ϕ∈(0,π), n=10, γ=0.75,0.95 and 1−α=0.90,0.99. (a) γ=0.75, (b) γ=0.95.

Based on the results in Table 2, we conduct a comparison between TBEa and UVa in Figure 2 with n=10, γ=0.75,0.95 and 1−α=0.90,0.99. The $\phi^{I}$‐values for Type I bands are utilized as ϕ‐axis. Figure 2 reveal that the r‐ratio is below 1 for large ϕ and 1−α (e.g., ϕ>2.8 and 1−α=0.99). Therefore, we can conclude that TBEa is better than UVa when both ϕ and 1−α are large, and n is small. It is also noteworthy that $\phi^{I}$ and $\phi^{II}$ are larger than 3 when s≥10, indicating that the x‐values of the training dataset are dispersed. Hence, asymmetric Type I bands should be used only for the training dataset having dispersed x‐values, a relatively large confidence level 1−α, and a small sample size n. Therefore, asymmetric Type II bands, like UVa, are recommended in general.

## 4 Illustrative Example

In order to illustrate how to determine the shelf‐life of a drug based on the method of SCBs for a percentile line, we revisit the real data example on drug stability from Ruberg and Hsu [17]. We also provide a visual demonstration of the SCBs and their corresponding constrained regions $R_{T}$. For the data from the first batch of Experiment One in Ruberg and Hsu [17], the fitted model is $ŷ=98.244-1.515(x-\bar{x})$ with $\bar{x}=\sum_{i=1}^{n}x_{i}/n=1.482$, $\hat{\sigma }=0.137$ and n=9.

For given y, X, (a,b)=(0,2), γ=0.05 and 1−α=0.95, Table 3 shows the r‐ratios of areas of regions $R_{T}$ relative to the UVa band, including (i) the symmetric SCBs (SB, TBU, TBE, V, TT, UV) and (ii) the asymmetric SCBs (SBa, TBUa, TBEa, Va, TTa, UVa). It is clear that the $\text{Area}(R_{T})$‐values for asymmetric SCBs are substantially smaller than those for symmetric SCBs under the MACS criterion. Based on the numerical results, we can conclude that the UVa band is the best under the MACS criterion in this example.

**TABLE 3:** Ratios r of $\text{Area}(R_{T})$ relative to the UVa band for the drug stability data.

Bands	Ratio	Bands	Ratio	Bands	Ratio	Bands	Ratio
$\frac{\text{SB}}{\text{UVa}}$	1.579	$\frac{V}{\text{UVa}}$	1.425	$\frac{\text{SBa}}{\text{UVa}}$	1.089	$\frac{\text{Va}}{\text{UVa}}$	1.002
$\frac{\text{TBU}}{\text{UVa}}$	1.473	$\frac{\text{TT}}{\text{UVa}}$	1.334	$\frac{\text{TBUa}}{\text{UVa}}$	1.089	$\frac{\text{TTa}}{\text{UVa}}$	1.000
$\frac{\text{TBE}}{\text{UVa}}$	1.235	$\frac{\text{UV}}{\text{UVa}}$	1.335	$\frac{\text{TBEa}}{\text{UVa}}$	1.091	$\frac{\text{UVa}}{\text{UVa}}$	1

*Note:* SB, TBU, TBE, V, TT and UV are the symmetric SCBs. SBa, TBUa, TBEa, Va, TTa and UVa are the asymmetric SCBs.

**FIGURE 3:**
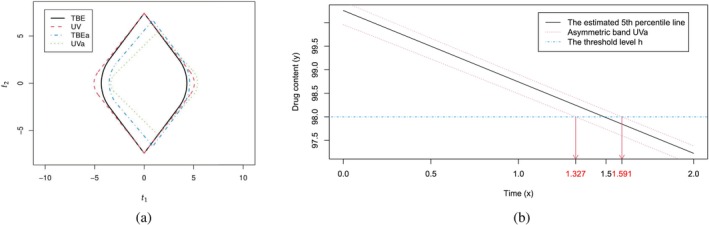
(a) The areas of $R_{T}$ for TBE, UV, TBEa, and UVa in the coordinates ($t_{1}$, $t_{2}$); (b) The 95% asymmetric band UVa for the 5th percentile.

In order to visually demonstrate the disparity in the areas of constrained regions $R_{T}$, we consider the $\text{Area}(R_{T})$ for TBE, UV, TBEa, and UVa, since TBE and UV represent the best options among Type I and Type II bands, respectively. Figure 3a gives the areas of $R_{T}$ for TBE, UV, TBEa, and UVa using a solid line, dash line, dash‐dotted line, and dotted line, respectively. It is shown in Figure 3a that $\text{Area}(R_{T})$ for UVa is the smallest.

In order to illustrate the estimation of shelf‐life based on SCBs for percentiles in linear regression, the UVa band is used, as it has been identified as the optimal choice from Table 3 and Figure 3a. In Figure 3b, the estimated percentile line $x^{T}\hat{\beta }+z_{\gamma }\hat{\sigma }/\xi_{1}$ with $\xi_{1}=\sqrt{\frac{2}{\nu }}\frac{\Gamma (\frac{\nu +1}{2})}{\Gamma (\frac{\nu }{2})}=0.965$ is shown by the solid line, and the UVa band is given by the dotted lines. The critical constants ($c_{1},\;c_{2}$) for UVa are (3.230, 2.016). For the given threshold h=98, which is given by the dash‐dotted line, one can infer from the UVa band that, the percentile line $x^{T}\beta +z_{\gamma }\sigma$ is above h before the time point x=1.327 with 1−α=95% confidence level, and so at least 1−γ=95% proportion of all the dosage units have drug content above h by this time point. But beyond the time point x=1.591, the percentile line $x^{T}\beta +z_{\gamma }\sigma$ is below h with 95% confidence level, and so less than 1−γ=95% proportion of all the dosage units have drug content above h. It should be noted that the true shelf‐life $X_{\gamma }$ at which $\beta_{0}+\beta_{1}(X_{\gamma }-\bar{x})+z_{\gamma }\sigma =h$ can be anywhere in the interval $X_{\gamma }\in (1.327,\;1.591)$.

In general, according to the area of $R_{T}$ in Figure 3a and the numerical results in Table 3, the asymmetric Type II bands (Va, UVa and TTa) have smaller volume of confidence sets for unknown parameter $\theta ={\left(\beta_{0},\;\beta_{1},\;\sigma \right)}^{T}$ and should be used under the MACS criterion.

## 5 Conclusion

Drug stability studies are an important part of any pharmaceutical drug development programme. The interval estimation of true shelf‐life based on the mean drug content of a selected dosage unit only guarantees that half of all the dosage units have the drug content no less than the pre‐specified level h by the estimated shelf‐life. This may not be satisfactory from the patients' point of view unless the therapeutic effect of a dosage unit with drug content below h is well understood.

In this paper, the true shelf‐life is set as the time point at which no more than 100γ% of all the dosage units will have the drug content less than a pre‐specified lowest acceptable limit h, where γ is a pre‐specified number close to 0. Constructing the exact (1−α)‐level SCBs for the 100γth percentile line in linear regression is the appropriate way for estimating the shelf‐life in this situation.

In this paper, we propose the MACS criterion, allowing for the comparison of different types of SCBs for percentile lines based on the area of the region for our new pivotal quantity T. In addition, our newly proposed method of computing the critical constants of SCBs for percentile lines is more efficient than the methods previously available in the statistical literature. Moreover, the optimal SCB under the MACS can be used to construct the interval estimation of shelf‐life in drug stability studies.

Based on our numerical results, we observe that the optimal MACS asymmetric bands are inherently superior to the corresponding symmetric bands under the MACS criterion, as expected, making them the preferred choice. In most cases, the asymmetric Type II bands perform better than the asymmetric Type I bands. Therefore, asymmetric Type II bands, like UVa, are recommended.

Although this paper only focuses on the simple linear regression model, the proposed method can be extended to multiple regression and polynomial regression. For multiple regression involving more than one covariate, the MACS‐based method can be applied, and the polar coordinates of K can be directly used to calculate the critical constants. The computational cost remains comparable to that of simple linear regression, taking approximately 10 s for symmetric SCBs and about 200 s for asymmetric SCBs. The computation time does not increase with the number of covariates. For polynomial regression, the critical constants cannot be determined directly using the polar coordinates of the pivotal quantity K. In such cases, a simulation‐based approach can be employed instead. The computation time increases due to the optimization involved. With 1 000 000 simulations, the computation time is approximately $3\times 10^{3}$ s for symmetric SCBs and over $2\times 10^{4}$ s for asymmetric SCBs. These times can be substantially reduced through parallel computing with an increased number of cores.

Furthermore, the MACS criterion can be used to select optimal simultaneous tolerance intervals for linear regression, which is currently under research and will be reported separately in the future.

## Conflicts of Interest

The authors declare no conflicts of interest.

## Supporting information




**Data S1.** The Supporting Information provides the computational costs of our newly proposed method and the previous simulation‐based method, as well as the additional numerical results in Section 3.

## Data Availability

The R code for the simulation studies and the real data example is available at https://github.com/Lingjiao‐WANG/MACS; the results of these analyses are presented in the paper and the Supporting Information.

## Appendix A Derivation of the confidence set $C_{\theta }$

To derive the confidence set $C_{\theta }$ for unknown parameters $\theta ={\left(\beta_{0},\;\beta_{1},\;\sigma \right)}^{T}$ according to the region $R_{K}$, we need identify the relation between θ and the pivotal quantity K. Recall $a={\left(\frac{z_{\gamma }\sqrt{n}}{\xi_{1}\sqrt{1+nz_{\gamma }^{2}\xi_{2}}},\;0\right)}^{T}$, $b={\left({\hat{\beta }}_{0},\;{\hat{\beta }}_{1},\;0\right)}^{T}$, $M=\left(\begin{array}{ll} \sqrt{\frac{1}{1+nz_{\gamma }^{2}\xi_{2}}} & 0\\ 0 & 1 \end{array}\right)$ and $U=\frac{1}{\hat{\sigma }}\left(\begin{array}{lll} \sqrt{n} & 0 & z_{\gamma }\sqrt{n}\\ 0 & \sqrt{S_{xx}} & 0 \end{array}\right)$ as defined in (7), we have

$$\begin{array}{ll} K & =\left(\begin{array}{c} K_{1}\\ K_{2} \end{array}\right)=\left(\begin{array}{c} \frac{N_{1}-z_{\gamma }\sqrt{n}}{U\sqrt{1+nz_{\gamma }^{2}\xi_{2}}}+\frac{z_{\gamma }\sqrt{n}}{\xi_{1}\sqrt{1+nz_{\gamma }^{2}\xi_{2}}}\\ N_{2}/U \end{array}\right)\\ & =\left(\begin{array}{c} \frac{N_{1}-z_{\gamma }\sqrt{n}}{U\sqrt{1+nz_{\gamma }^{2}\xi_{2}}}\\ N_{2}/U \end{array}\right)+\left(\begin{array}{c} \frac{z_{\gamma }\sqrt{n}}{\xi_{1}\sqrt{1+nz_{\gamma }^{2}\xi_{2}}}\\ 0 \end{array}\right)\\ & =\frac{1}{\hat{\sigma }}\left(\begin{array}{c} \frac{\sqrt{n}(\hat{\beta_{0}}-\beta_{0})-\sigma z_{\gamma }\sqrt{n}}{\sqrt{1+nz_{\gamma }^{2}\xi_{2}}}\\ \sqrt{S_{xx}}(\hat{\beta_{1}}-\beta_{1}) \end{array}\right)+a\\ & =\frac{1}{\hat{\sigma }}\left(\begin{array}{cc} \sqrt{\frac{1}{1+nz_{\gamma }^{2}\xi_{2}}} & 0\\ 0 & 1 \end{array}\right)\left(\begin{array}{ccc} \sqrt{n} & 0 & z_{\gamma }\sqrt{n}\\ 0 & \sqrt{S_{xx}} & 0 \end{array}\right)\left(\begin{array}{c} \hat{\beta_{0}}-\beta_{0}\\ \hat{\beta_{1}}-\beta_{1}\\ -\sigma \end{array}\right)+a\\ & =MU(b-\theta )+a. \end{array}$$

Hence, the confidence set $C_{\theta }$ can be expressed as

$$\begin{array}{ll} C_{\theta } & =\left\{\theta :\;K=MU(b-\theta )+a\in R_{K}\right\}\\ & =\left\{\theta :\;MU(b-\theta )+a\in R_{K}\right\}. \end{array}$$

## Appendix B Derivation of $\text{Area}(R_{K})$

Set $c_{min}=\min(c_{1},\;c_{2})$ and $c_{max}=\max(c_{1},\;c_{2})$, where $c_{1}$ and $c_{2}$ are defined in (4). The $\text{Area}(R_{K})$ in (8) can be calculated for the following situations.

1. When ϕ<π/2, $\text{Area}(R_{K})={\left(c_{min}/\cos\phi +c_{max}\right)}^{2}/\tan\phi -c_{min}^{2}\tan\phi +\phi (c_{1}^{2}+c_{2}^{2})/2.$
2. When ϕ=π/2, $\text{Area}(R_{K})=2c_{1}c_{2}+\phi (c_{1}^{2}+c_{2}^{2})/2.$
3. When ϕ>π/2,
  (1) if $c_{min}/\cos(\pi -\phi )>c_{max}$, $\text{Area}(R_{K})=c_{min}^{2}\tan(\pi -\phi )-{\left(c_{min}/\cos(\pi -\phi )-c_{max}\right)}^{2}/\tan(\pi -\phi )+\phi (c_{1}^{2}+c_{2}^{2})/2$;
  (2) if $c_{min}/\cos(\pi -\phi )\leq c_{max}$, $\text{Area}(R_{K})=c_{min}\sqrt{c_{max}^{2}-c_{min}^{2}}+c_{min}^{2}\phi /2+c_{max}^{2}(2\pi -\phi -2\arccos(c_{min}/c_{max}))/2$.

## Appendix C Joint density function of $\left(K_{1},K_{2}\right)$

Since $N_{1}∼N(0,\;1)$, $N_{2}∼N(0,\;1)$ and $U∼\sqrt{\chi_{\nu }^{2}/\nu }$ with ν=n−2 in (4) are independent, the joint density function of $\left(N_{1},\;N_{2},\;U\right)$ is

(A1)
$$f(n_{1},n_{2},u)=\frac{\nu^{\nu /2}}{2^{\nu /2}\pi \Gamma (\nu /2)}u^{\nu -1}\exp(-\frac{1}{2}(n_{1}^{2}+n_{2}^{2}+\nu u^{2})).$$

Now, consider the pdf for $K={\left(K_{1},\;K_{2}\right)}^{T}$ with

$$K_{1}=q_{1}\frac{N_{1}-q_{3}}{U}+q_{2}\;\text{and}\;K_{2}=N_{2}/U$$

where $q_{1}=\sqrt{\frac{1}{1+nz_{\gamma }^{2}\xi_{2}}}$, $q_{2}=\frac{z_{\gamma }\sqrt{n}}{\xi_{1}\sqrt{1+nz_{\gamma }^{2}\xi_{2}}}$ and $q_{3}=z_{\gamma }\sqrt{n}$. Define the random variable $K_{3}=U$, so that $N_{1}$, $N_{2}$ and U can be solved in terms of $K_{1}$, $K_{2}$ and $K_{3}$ as

(A2)
$$N_{1}=K_{3}\frac{K_{1}-q_{2}}{q_{1}}+q_{3},\;N_{2}=K_{2}K_{3}\;\text{and}\;U=K_{3}.$$

This gives the following Jacobian matrix and determinant

(A3)
$$\begin{array}{ll} J & =\left(\begin{array}{ccc} \frac{\partial N_{1}}{\partial K_{1}} & \frac{\partial N_{1}}{\partial K_{2}} & \frac{\partial N_{1}}{\partial K_{3}}\\ \frac{\partial N_{2}}{\partial K_{1}} & \frac{\partial N_{3}}{\partial K_{2}} & \frac{\partial N_{3}}{\partial K_{3}}\\ \frac{\partial U}{\partial K_{1}} & \frac{\partial U}{\partial K_{2}} & \frac{\partial U}{\partial K_{3}} \end{array}\right)=\left(\begin{array}{ccc} \frac{K_{3}}{q_{1}} & 0 & \frac{K_{1}-q_{2}}{q_{1}}\\ 0 & K_{3} & K_{2}\\ 0 & 0 & 1 \end{array}\right)\\ \left|J\right| & =K_{3}^{2}/q_{1}. \end{array}$$

It follows immediately that the joint density of $K_{1}$, $K_{2}$ and $K_{3}$ is given by

$$f_{K_{1},K_{2},K_{3}}(k_{1},k_{2},k_{3})=f_{N,U}(n_{1},n_{2},u)|J|.$$

Combining ((A1), (A2), (A3)) gives

$$\begin{array}{ll} f_{K_{1},K_{2},K_{3}}(k_{1},k_{2},k_{3}) & =\frac{\nu^{\nu /2}}{q_{1}2^{\nu /2}\pi \Gamma (\nu /2)}k_{3}^{\nu +1}\\ & \;\times \exp\left\{-\frac{1}{2}\left[\left({\left(\frac{k_{1}-q_{2}}{q_{1}}\right)}^{2}+k_{2}^{2}+\nu \right)k_{3}^{2}\right.\right.\\ & \;\left.\left.+\frac{2q_{3}(k_{1}-q_{2})}{q_{1}}k_{3}+q_{3}^{2}\right]\right\}. \end{array}$$

Integrating out $k_{3}$ gives the (marginal) density of $K_{1}$ and $K_{2}$

$$\begin{array}{ll} f_{K_{1},K_{2}}(k_{1},k_{2}) & =\frac{\exp(-q_{3}^{2}/2)\nu^{\nu /2}}{q_{1}2^{\nu /2}\pi \Gamma (\nu /2)}\int_{0}^{\infty }k_{3}^{\nu +1}\\ & \;\times \exp\left\{-\frac{1}{2}\left[\left({\left(\frac{k_{1}-q_{2}}{q_{1}}\right)}^{2}+k_{2}^{2}+\nu \right)k_{3}^{2}\right.\right.\\ & \;\left.\left.+\frac{2q_{3}(k_{1}-q_{2})}{q_{1}}k_{3}\right]\right\}dk_{3}. \end{array}$$

## Appendix D Proof of Theorem 2

Before we prove the Theorem 2, we need the following two lemmas.

Lemma 1
*Let* K *be the pivotal quantity in* (*5*) *and* $R_{K}$ *be the region in* (*6*). *For given critical constants* $c_{1}$ *and* $c_{2}$, *the confidence level of the SCB in* (*3*) *is*

$$\begin{array}{ll} & P\left\{x^{T}\hat{\beta }+z_{\gamma }\hat{\sigma }/\xi_{1}-c_{1}\hat{\sigma }\sqrt{x^{T}{\left(X^{T}X\right)}^{-1}x+{\left(z_{\gamma }\right)}^{2}\xi_{2}}\leq x^{T}\beta +z_{\gamma }\sigma \right.\\ & \left.\;\leq x^{T}\hat{\beta }+z_{\gamma }\hat{\sigma }/\xi_{1}+c_{2}\hat{\sigma }\sqrt{x^{T}{\left(X^{T}X\right)}^{-1}x+{\left(z_{\gamma }\right)}^{2}\xi_{2}}\;\text{for all}\;x\in (a,b)\right\}\\ & \;=P\left\{-c_{2}\leq \frac{w^{T}K}{\left\|w\right\|}\leq c_{1}\;\text{for all}\;x\in (a,b)\right\}\\ & \;=P\left\{K\in R_{K}\right\}. \end{array}$$

In order to calculate the probability $P\{K\in R_{K}\}$, all three different situations are considered below. The angles $\phi_{1}$ and $\phi_{2}$ are defined in Figure 1a,b, while the angles $\zeta_{1}$, $\zeta_{2}$, $\phi_{1}^{\prime }$, $\phi_{1}^{\prime \prime }$, $\phi_{2}^{\prime }$ and $\phi_{2}^{\prime \prime }$ are defined in Figure 1d.

1. When $\phi <\frac{\pi }{2}$, we have $\zeta_{1}=\arctan\left[\left(\frac{c_{2}}{\cos\phi }+c_{1}\right)\tan\left(\frac{\pi }{2}-\phi \right)/c_{1}\right]$, $\zeta_{2}=\pi -\zeta_{1}-\phi$, $\phi_{1}^{\prime }=\phi_{1}^{\prime \prime }=\phi_{1}$ and $\phi_{2}^{\prime }=\phi_{2}^{\prime \prime }=\phi_{2}$.
2. When $\phi =\frac{\pi }{2}$, we have $\zeta_{1}=\arctan\left(\frac{c_{2}}{c_{1}}\right)$, $\zeta_{2}=\pi -\zeta_{1}-\phi$, $\phi_{1}^{\prime }=\phi_{1}^{\prime \prime }=\phi_{1}$ and $\phi_{2}^{\prime }=\phi_{2}^{\prime \prime }=\phi_{2}$.
3. When $\phi >\frac{\pi }{2}$,
  (1) if $c_{min}/\cos(\pi -\phi )>c_{max}$, we have $\zeta_{1}=\arctan\left[\left(\frac{c_{2}}{\cos(\pi -\phi )}-c_{1}\right)\tan\left(\phi -\frac{\pi }{2}\right)/c_{1}\right]$, $\zeta_{2}=\pi -\zeta_{1}-\phi$, $\phi_{1}^{\prime }=\phi_{1}^{\prime \prime }=\phi_{1}$ and $\phi_{2}^{\prime }=\phi_{2}^{\prime \prime }=\phi_{2}$;
  (2) if $c_{min}/\cos(\pi -\phi )\leq c_{max}$,
    (i) suppose $c_{1}\leq c_{2}$, $\zeta_{1}=\arccos\left(\frac{c_{1}}{c_{2}}\right)$, $\zeta_{2}=0$, $\phi_{1}^{\prime }=\phi_{1}$, $\phi_{2}^{\prime }=\phi_{2}$, $\phi_{1}^{\prime \prime }=\pi -\phi_{2}^{\prime }-\zeta_{1}$, and $\phi_{2}^{\prime \prime }=\pi -\phi_{1}^{\prime }-\zeta_{1}$;
    (ii) suppose $c_{1}>c_{2}$, $\zeta_{1}=0$, $\zeta_{2}=\arccos\left(\frac{c_{2}}{c_{1}}\right)$, $\phi_{1}^{\prime \prime }=\phi_{1}$, $\phi_{2}^{\prime \prime }=\phi_{2}$, $\phi_{1}^{\prime }=\pi -\phi_{2}^{\prime \prime }-\zeta_{1}$, and $\phi_{2}^{\prime }=\pi -\phi_{1}^{\prime \prime }-\zeta_{1}$.

The next lemma provides the derivation of $R_{K}$ using a method involving partitions and polar coordinates (R,δ).

Lemma 2
*For any SCB in* (*3*), *the corresponding* $R_{K}$ *in Figure* *1* *d is partitioned as*:

$$R_{K}={⋃}_{i=1}^{4}\left(R_{K;M_{i}}\cup R_{K;O_{i}}\right)$$

*where*

$$\begin{array}{ll} R_{K;M_{1}} & =\{K:\;\delta \in [\phi_{1}^{\prime },\;\phi_{1}^{\prime }+\zeta_{1}),\;0\leq \left(\cos\left(\phi_{1}^{\prime }\right),\;\sin\left(\phi_{1}^{\prime }\right)\right)K\leq c_{1}\}\\ & =\left\{(R,\;\delta ):\;\delta \in [\phi_{1}^{\prime },\;\phi_{1}^{\prime }+\zeta_{1}),\;R\leq \frac{c_{1}}{\cos\left(\delta -\phi_{1}^{\prime }\right)}\right\}\\ R_{K;M_{2}} & =\{K:\;\delta \in [\phi_{1}^{\prime }+\zeta_{1},\;\pi -\phi_{2}^{\prime \prime }),\;0\leq \left(\cos\left(\pi -\phi_{2}^{\prime \prime }\right),\;\sin\left(\pi -\phi_{2}^{\prime \prime }\right)\right)K\leq c_{2}\}\\ & =\left\{(R,\;\delta ):\;\delta \in [\phi_{1}^{\prime }+\zeta_{1},\;\pi -\phi_{2}^{\prime \prime }),\;R\leq \frac{c_{2}}{\cos\left(\delta -\pi +\phi_{2}^{\prime \prime }\right)}\right\}\\ R_{K;M_{3}} & =\{K:\;\delta \in [\pi +\phi_{1}^{\prime \prime },\;\pi +\phi_{1}^{\prime \prime }+\zeta_{2}),\;0\leq \left(\cos\left(\pi +\phi_{1}^{\prime \prime }\right),\;\sin\left(\pi +\phi_{1}^{\prime \prime }\right)\right)K\leq c_{2}\}\\ & =\left\{(R,\;\delta ):\;\delta \in [\pi +\phi_{1}^{\prime \prime },\;\pi +\phi_{1}^{\prime \prime }+\zeta_{2}),\;R\leq \frac{c_{2}}{\cos\left(\delta -\pi -\phi_{1}^{\prime \prime }\right)}\right\}\\ R_{K;M_{4}} & =\{K:\;\delta \in [\pi +\phi_{1}^{\prime \prime }+\zeta_{2},\;2\pi -\phi_{2}^{\prime }),\;0\leq \left(\cos\left(2\pi -\phi_{2}^{\prime }\right),\;\sin\left(2\pi -\phi_{2}^{\prime }\right)\right)K\leq c_{1}\}\\ & =\left\{(R,\;\delta ):\;\delta \in [\pi +\phi_{1}^{\prime \prime }+\zeta_{2},\;2\pi -\phi_{2}^{\prime }),\;R\leq \frac{c_{1}}{\cos\left(\delta -2\pi +\phi_{2}^{\prime }\right)}\right\}\\ R_{K;O_{1}} & =\{K:\;\delta \in [0,\;\phi_{1}^{\prime }),\;||K||\leq c_{1}\}=\{(R,\;\delta ):\;\delta \in [0,\;\phi_{1}^{\prime }),\;R\leq c_{1}\}\\ R_{K;O_{2}} & =\{K:\;\delta \in [\pi -\phi_{2}^{\prime \prime },\;\pi ),\;||K||\leq c_{2}\}=\{(R,\;\delta ):\;\delta \in [\pi -\phi_{2}^{\prime \prime },\;\pi ),\;R\leq c_{2}\}\\ R_{K;O_{3}} & =\{K:\;\delta \in [\pi ,\;\pi +\phi_{1}^{\prime \prime }),\;||K||\leq c_{2}\}=\{(R,\;\delta ):\;\delta \in [\pi ,\;\pi +\phi_{1}^{\prime \prime }),\;R\leq c_{2}\}\\ R_{K;O_{4}} & =\{K:\;\delta \in [2\pi -\phi_{2}^{\prime },\;2\pi ),\;||K||\leq c_{1}\}=\{(R,\;\delta ):\;\delta \in [2\pi -\phi_{2}^{\prime },\;2\pi ),\;R\leq c_{1}\}. \end{array}$$

From Lemmas 1 and 2, we can derive Theorem 2.

Following Lemmas 1 and 2, the confidence level of the SCB in (3) given $c_{1}$ and $c_{2}$ is

(A4)
$$\begin{array}{ll} & P\left\{x^{T}\hat{\beta }+z_{\gamma }\hat{\sigma }⁄\xi_{1}-c_{1}\hat{\sigma }\sqrt{x^{T}{\left(X^{T}X\right)}^{-1}x+{\left(z_{\gamma }\right)}^{2}\xi_{2}}\leq x^{T}\beta +z_{\gamma }\sigma \right.\\ & \;\left.\leq x^{T}\hat{\beta }+z_{\gamma }\hat{\sigma }⁄\xi_{1}+c_{2}\hat{\sigma }\sqrt{x^{T}{\left(X^{T}X\right)}^{-1}x+{\left(z_{\gamma }\right)}^{2}\xi_{2}}\;\;\text{for}\text{all}\;x\in (a,\;b)\right\}\\ & \;=P\left\{-c_{2}\leq \frac{w^{T}K}{\left\|w\right\|}\leq c_{1}\;\text{for all}\;x\in (a,\;b)\right\}\\ & \;=P\left\{K\in R_{K}\right\}\\ & \;=P\left\{K\in {⋃}_{i=1}^{4}\left(R_{K;M_{i}}\cup R_{K;O_{i}}\right)\right\}\\ & \;=P\left\{(R,\;\delta )\in {⋃}_{i=1}^{4}\left(R_{K;M_{i}}\cup R_{K;O_{i}}\right)\right\}\\ & \;=\sum_{i=1}^{4}\left(P\left\{(R,\;\delta )\in R_{K;M_{i}}\right\}+P\left\{(R,\;\delta )\in R_{K;O_{i}}\right\}\right)\\ & \;=\sum_{i=1}^{4}\left(\int \int_{(R,\;\delta )\in R_{K;M_{i}}}f_{R,\delta }(r,\delta )drd\delta \right.\left.+\int \int_{(R,\;\delta )\in R_{K;O_{i}}}f_{R,\delta }(r,\delta )drd\delta \right). \end{array}$$

The Equation (A4) is based on the independence of regions $R_{K;M_{i}}$ and $R_{K;O_{i}}$, i=1,…,4.
