# Supplementary material for: Minimum Area Confidence Set Optimality for Simultaneous Confidence Bands for Percentiles With Applications to Drug Shelf‐Life Estimation
Source: Stat Med. 2025 Sep 9;44(20-22):e70184. doi: 10.1002/sim.70184 (PMC12418920; doi:10.1002/sim.70184)
Supplement: Supplementary file 1 — Data S1. The Supporting Information provides the computational costs of our newly proposed method and the previous simulation‐based method, as well as the additional numerical results in Section 3. [file SIM-44-0-s001.pdf]

Supplementary Material for the Paper "Minimum Area Confidence Set Optimality for Simultaneous Confidence Bands for Percentiles with Applications to Drug Shelf-Life Estimation"

Lingjiao Wang[1], Yang Han[1*], Wei Liu[2], Frank Bretz[3]

[1]Department of Mathematics, University of Manchester, Manchester, UK

[2]School of Mathematical Sciences and Southampton Statistical Sciences Research Institute, University of Southampton, Southampton, UK

[3]Novartis Pharma AG, Basel, Switzerland

*Email: yang.han@manchester.ac.uk

This document provides supplementary material to the main text. It presents the detailed computational costs (in seconds) of our newly proposed method and the previous simulation-based method, as well as the additional simulation results in Section 3.

A. Computational Costs

From our numerical investigations, the computational costs for determining the critical constants are presented in Tables 1 and 2. Using our new method, it takes no more than 2×10^1 seconds for symmetric SCBs and less than 2×10^2 seconds for asymmetric SCBs, when $n = 10$, on an ordinary Windows PC (Intel(R) Core(TM) i7-6700 CPU with 3.40GHz, 3.41 GHz, RAM 16.0 GB). When $n = 100$, the computation time is no more than 2×10^2 seconds for symmetric SCBs and less than 8×10^2 seconds for asymmetric SCBs in most cases. In comparison, the simulation-based method in Han *et al.* (2015) requires more than 8×10^2 seconds for symmetric SCBs and no less than 4×10^3 seconds for asymmetric SCBs on the same Windows PC, using 1,000,000 simulations. Overall, our new method appears significantly more efficient.

Table 1. The computational costs (in seconds) for symmetric bands, using our new method (NEW) and the simulation-based method (HAN) by Han *et al.* (2015).

$1-\alpha$	γ	n	SB		TBU		TBE		V		TT		UV	
			NEW	HAN	NEW	HAN	NEW	HAN	NEW	HAN	NEW	HAN	NEW	HAN
0.9	0.75	10	1×10^1	9×10^2	1×10^1	9×10^2	1×10^1	9×10^2	1×10^1	9×10^2	1×10^1	9×10^2	1×10^1	9×10^2
		100	2×10^1	9×10^2	3×10^1	9×10^2	3×10^1	9×10^2	2×10^1	9×10^2	3×10^1	9×10^2	3×10^1	9×10^2
	0.95	10	1×10^1	8×10^2	1×10^1	9×10^2	1×10^1	9×10^2	1×10^1	9×10^2	1×10^1	9×10^2	1×10^1	9×10^2
		100	8×10^1	8×10^2	5×10^1	8×10^2	8×10^1	8×10^2	8×10^1	8×10^2	6×10^1	8×10^2	6×10^1	8×10^2
0.99	0.75	10	1×10^1	9×10^2	1×10^1	1×10^3	1×10^1	1×10^3	1×10^1	1×10^3	1×10^1	1×10^3	1×10^1	1×10^3
		100	3×10^1	9×10^2	3×10^1	9×10^2	3×10^1	9×10^2	3×10^1	1×10^3	4×10^1	9×10^2	4×10^1	1×10^3
	0.95	10	2×10^1	9×10^2	1×10^1	1×10^3	1×10^1	1×10^3	1×10^1	1×10^3	1×10^1	1×10^3	1×10^1	1×10^3
		100	3×10^2	1×10^3	2×10^2	1×10^3	2×10^2	1×10^3	2×10^2	1×10^3	1×10^2	9×10^2	1×10^2	9×10^2

Table 2. The computational costs (in seconds) for asymmetric bands, using our new method (NEW) and the simulation-based method (HAN) by Han *et al.* (2015).

$1-\alpha$	γ	n	SBa		TBUa		TBEa		Va		TTa		UVa	
			NEW	HAN	NEW	HAN	NEW	HAN	NEW	HAN	NEW	HAN	NEW	HAN
0.9	0.75	10	2×10^2	3×10^4	1×10^2	3×10^4	2×10^2	3×10^4	1×10^2	3×10^4	2×10^2	3×10^4	1×10^2	3×10^4
		100	2×10^2	3×10^4	2×10^2	3×10^4	3×10^2	3×10^4	2×10^2	3×10^4	3×10^2	3×10^4	3×10^2	3×10^4
	0.95	10	1×10^2	3×10^4	2×10^2	3×10^4	2×10^2	3×10^4	1×10^2	3×10^4	1×10^2	3×10^4	1×10^2	3×10^4
		100	8×10^2	3×10^4	8×10^2	3×10^4	5×10^2	3×10^4	7×10^2	3×10^4	4×10^2	3×10^4	4×10^2	3×10^4
0.99	0.75	10	2×10^2	5×10^3	2×10^2	5×10^3	2×10^2	5×10^3	2×10^2	5×10^3	2×10^2	5×10^3	2×10^2	5×10^3
		100	3×10^2	5×10^3	3×10^2	5×10^3	3×10^2	5×10^3	3×10^2	5×10^3	3×10^2	5×10^3	3×10^2	5×10^3
	0.95	10	2×10^2	5×10^3	2×10^2	5×10^3	2×10^2	5×10^3	2×10^2	5×10^3	2×10^2	5×10^3	2×10^2	5×10^3
		100	4×10^3	5×10^3	3×10^3	5×10^3	2×10^3	5×10^3	1×10^3	4×10^3	8×10^2	5×10^3	1×10^3	5×10^3

B. Additional Simulation Results in Section 3

In this section, we assess the performance of symmetric and asymmetric SCBs within each type (Type I bands with $\xi = 0$ and Type II bands with $\xi \neq 0$). Table 3 presents the r-ratios of Area($\boldsymbol{R_T}$) for symmetric and asymmetric Type I bands (SB, TBU, TBE, SBa, TBUa, TBEa) relative to the TBEa band, while Table 4 shows the r-ratios of Area($\boldsymbol{R_T}$) for symmetric and asymmetric Type II bands (V, TT, UV, Va, TTa, UVa) relative to the UVa band.

Table 3. Ratios r, relative to TBEa, of Area($\boldsymbol{R_T}$) for symmetric and asymmetric Type I SCBs: SB, TBU, TBE, SBa, TBUa.

$1-\alpha$	γ	n	s	ϕ^I	SB	TBU	TBE	SBa	TBUa	TBEa
0.9	0.75	10	0.1	0.613	1.038	1.020	1.000	1.000	1.000	1
			1	2.529	1.039	1.024	1.003	1.001	1.000	1
			10	3.078	1.040	1.025	1.004	0.999	0.999	1
		100	0.1	1.571	1.003	1.003	1.002	1.000	1.000	1
			1	2.942	1.002	1.001	1.000	1.000	1.000	1
			10	3.122	1.002	1.001	1.000	0.999	0.999	1
	0.95	10	0.1	0.613	1.158	1.079	1.000	0.999	0.999	1
			1	2.529	1.159	1.088	1.005	0.970	0.980	1
			10	3.078	1.168	1.097	1.013	0.941	0.957	1
		100	0.1	1.571	1.006	1.003	1.000	0.999	1.000	1
			1	2.942	1.006	1.003	1.001	0.990	0.994	1
			10	3.122	1.010	1.007	1.004	0.986	0.991	1
0.99	0.75	10	0.1	0.613	1.322	1.287	1.206	1.000	1.000	1
			1	2.529	1.248	1.223	1.164	1.005	1.003	1
			10	3.078	1.246	1.221	1.163	1.010	1.007	1
		100	0.1	1.571	1.025	1.021	1.014	1.000	1.000	1
			1	2.942	1.025	1.021	1.015	0.999	1.000	1
			10	3.122	1.026	1.023	1.017	0.999	0.999	1
	0.95	10	0.1	0.613	1.807	1.731	1.554	1.000	1.000	1
			1	2.529	1.936	1.858	1.677	0.971	0.978	1
			10	3.078	2.063	1.979	1.786	0.992	0.990	1
		100	0.1	1.571	1.085	1.071	1.046	1.000	1.000	1
			1	2.942	1.115	1.100	1.075	0.985	0.990	1
			10	3.122	1.128	1.114	1.088	0.982	0.988	1

ϕ^I is the angle ϕ in (10) for Type I bands with $\xi = 0$ (SB, TBU, TBE, SBa, TBUa and TBEa).

Table 4. Ratios r, relative to UVa, of Area($\boldsymbol{R_T}$) for symmetric and asymmetric Type II SCBs: V, TT, UV, Va, TTa.

$1-\alpha$	γ	n	s	ϕ^{II}	V	TT	UV	Va	TTa	UVa
0.9	0.75	10	0.1	0.543	1.038	1.020	1.020	1.000	1.000	1
			1	2.451	1.036	1.022	1.023	1.001	1.000	1
			10	3.070	1.033	1.020	1.020	1.002	1.000	1
		100	0.1	1.466	1.002	1.001	1.001	1.000	1.000	1
			1	2.920	1.002	1.001	1.001	1.000	1.000	1
			10	3.119	1.002	1.001	1.001	1.000	1.000	1
	0.95	10	0.1	0.543	1.161	1.080	1.081	1.000	1.000	1
			1	2.451	1.139	1.089	1.090	1.002	0.999	1
			10	3.070	1.115	1.069	1.071	1.006	0.998	1
		100	0.1	1.466	1.006	1.003	1.003	1.000	1.000	1
			1	2.920	1.006	1.003	1.003	1.000	1.000	1
			10	3.119	1.006	1.003	1.003	1.000	1.000	1
0.99	0.75	10	0.1	0.543	1.322	1.286	1.286	1.000	1.000	1
			1	2.451	1.195	1.172	1.172	1.002	1.000	1
			10	3.070	1.164	1.143	1.143	1.003	0.999	1
		100	0.1	1.466	1.024	1.021	1.021	1.000	1.000	1
			1	2.920	1.018	1.015	1.015	1.000	1.000	1
			10	3.119	1.018	1.015	1.015	1.000	1.000	1
	0.95	10	0.1	0.543	1.807	1.730	1.731	1.000	1.000	1
			1	2.451	1.523	1.467	1.464	1.003	0.999	1
			10	3.070	1.370	1.320	1.317	1.005	0.997	1
		100	0.1	1.466	1.083	1.070	1.070	1.000	1.000	1
			1	2.920	1.056	1.046	1.046	1.001	1.000	1
			10	3.119	1.053	1.044	1.044	1.001	1.000	1

ϕ^{II} is the angle ϕ in (10) for Type II bands with $\xi \neq 0$ (V, TT, UV, Va, TTa and UVa).
